# Chief Executive Officer Collectivism and Corporate Pollution Abatement Behavior: Evidence From Industrial Firms in China

**DOI:** 10.3389/fpsyg.2022.946111

**Published:** 2022-07-06

**Authors:** Shumin Wang, Yikun Huang, Chao Zhong, Boxi Li

**Affiliations:** ^1^Research Center for Economy of Upper Reaches of the Yangtse River, Chongqing Technology and Business University, Chongqing, China; ^2^School of Public Affairs Administration, China Agricultural University, Beijing, China; ^3^Business School, Beijing Normal University, Beijing, China; ^4^China Resources Environment Carbon Neutrality Research Programme, Beijing, China

**Keywords:** environmental regulation, climate change, financing constraints, pollution reduction, CEO collectivism

## Abstract

This study examines the relationship between chief executive officers (CEOs)’ collectivistic cultural background and corporate pollution abatement behavior among industrial firms in China. Using hand-collected data on birthplaces of CEOs of the industrial firms, we provided robust evidence that CEOs born in provinces with a higher level of collectivistic culture promote corporate pollution abatement performance. This study further shows that firms exhibit significant differences in their emission reduction behavior when firms are subjected to environmental regulation shocks: firms with collectivistic CEOs tend to reduce more pollution than firms with individualistic CEOs without sacrificing their firms’ production.

## Introduction

The increased prominence of climate policy on government agendas worldwide has rekindled interest in the best design of large-scale environmental externality control. Climate change, also known as the “ultimate commons problem” (Stavins, 2011; Wan et al., 2021), is driven by anthropogenic greenhouse gas (GHG) emissions such as carbon dioxide (CO_2_) and is projected to have severe ecological and economic effects (Kumar, 2007). Globally, the industrial sector is a major source of greenhouse gas emissions. The industrial sector and primary industry contribute to over 40% of global GHG emissions (OECD et al., 2010). In 2000, total carbon emissions from the industrial sector were expected to be 60.3 MtC. Command-and-control strategies have long been the most prevalent form of environmental regulation in the industrial sector. Economists have historically favored market-based mechanisms such as taxes and tradable permit systems because they are more efficient in both static and dynamic terms (e.g., Montgomery, 1972; Milliman and Prince, 1989; Tietenberg, 1990).

To curb industrial firms’ pollution emissions in China, the Chinese government has been levying emission fees on these firms’ pollution emissions since 2003. Along with reducing firms’ pollutant emissions to a certain amount, the emission fee policy has brought certain negative consequences for firms and the whole economy. In the face of increasingly strict market regulation of emissions, such as the imposition of emission fees, firms typically have two options for reducing their emissions (Montero, 1998; Coria, 2009; Hatcher, 2012). The first strategy is pollution control, which reduces emissions. The second method is to reduce pollutant emissions by reducing firms’ production directly. However, reducing emissions through output reduction directly influences firms’ production and operations, resulting in significant macroeconomic swings (Bu and Liao, 2014; Li et al., 2016; Liu et al., 2020). This study investigates which companies are more likely to cut their pollution emissions and which are more likely to lower their production in response to the emission fee policy.

According to research, the collectivistic and individualistic cultural backgrounds of chief executive officers (CEOs) are associated with their pro-environmental behavior. According to available research, collectivism (as opposed to individualism) is defined by an interdependent self (as opposed to an independent self) (Markus and Kitayama, 1991). Specifically, collectivists are concerned with group norms and collective harmony, and they place group aims above their own (Wagner and Moch, 1986; Strunk and Chang, 1999; Voronov and Singer, 2002). Individualists value human autonomy and uniqueness and prioritize personal aspirations over group objectives. In this research, we argued that collectivistic CEOs are more likely than individualistic CEOs to take measures to reduce business pollution while maintaining company production in response to the change in environmental policy. This presumption is supported by the following arguments. Individualist vs. collectivist orientations have been demonstrated to influence environmentally conscious behavior. Individuals with collectivistic tendencies are more likely than those with individualistic tendencies to engage in a variety of pro-environmental behaviors, such as resource conservation and green shopping. Moreover, according to a poll performed in New Zealand by Semenova (2015), the more ecologically engaged group (representing sustainable communities) had a more collectivistic value orientation than the less environmentally active group. Environmental activists were more likely to embrace self-transcendent values (similar to collectivism, e.g., universalism-concern), but non-activists were more likely to endorse self-interest values (similarly to individualism, e.g., self-direction). Therefore, when confronted with stringent environmental regulations, collectivistic CEOs are presumed to take actions to directly reduce their firms’ pollution emissions without reducing production, whereas individualistic CEOs may choose to reduce production in order to reduce pollution emissions, resulting in the emission per unit of the product unchanged.

To investigate the effect of the CEO’s collectivistic background on corporate emission behaviors, we manually collected birthplace data for 9,227 out of 29,751 CEOs of our sample industrial firms from 2004 to 2013, which are then matched with the pollution information from Chinese Industrial Firm Pollution Emission (CIFPE) Database. We conducted empirical research utilizing the difference-in-difference method with the CEO birthplace information and the pollution data from industrial companies from 2004 to 2013 in response to the exogenous policy shock of the 2007 emission charge increase. Our primary measure of corporate pollution emissions is the annual emissions of both CO_2_ and sulfur dioxide (SO_2_) in tones. We found that CEOs born in provinces with a stronger collectivistic culture tend to reduce emissions. When controlling for firm characteristics, firms managed by such CEOs lower their pollution emissions and retain their production levels following an increase in the emission fee. While firms led by individualistic CEOs also lowered their total emissions after the rise in emission fees, the decreased emissions were accomplished by cutting production. On average, the emission per unit of output was reduced by 12.6% more for firms led by collectivistic CEOs than those led by individualistic CEOs.

This study makes the following three contributions to the existing literature. First, we complemented and broadened an emerging body of research that links culture to corporate behaviors and economic outcomes (e.g., Hilary and Hui, 2009; Ahern et al., 2015; DeBacker et al., 2015; Nguyen et al., 2018; Fitzgerald and Liu, 2020). Our study shows that CEOs’ individualistic cultural values from their hometowns shape corporate pollution abatement behaviors. Second, existing literature has examined the effects of pollution emission regulation on air pollution (Henderson, 1996; Greenstone, 2004), industrial activity (Becker and Henderson, 2000; Greenstone, 2002), plant births and deaths (Henderson, 1996; Levinson, 1996; List et al., 2003), plant productivity (Berman and Bui, 2001; Bu and Liao, 2021), and market structure (Bu et al., 2021). This is the first study to systematically evaluate the implementation of China’s emission fee policy change on industrial firms’ pollution control. Third, this study used a micro-matching sample at the firm level, which combined the Chinese Industrial Firm (CIF) and the Chinese Industrial Firm Pollution Emission (CIFPE) databases. The sample contained firm-related economic and financial indicators and a series of various pollutant emission indicators at the firm level. This provides the basis for reliable evidence for our study.

The subsequent sections of this study are organized as follows: the “Institutional Background” section and the “Hypothesis Development” section introduce the background of the emission fee policy and the agricultural root of collectivism in China and develop research hypotheses. The “Data” section and the “Empirical Analysis” section introduce the dataset and empirical analysis. The “Heterogeneous Analysis” section presents the heterogeneous analysis. The “Conclusion and Policy Implication” section concludes the whole study and makes policy implications.

## Institutional Background

### Emission Fee Policy

In the early 1970s, the pollution levy system was initially implemented in OECD nations. China first introduced the idea of a pollution levy system in 1978, after learning from the environmental management practices of Western countries. Subsequently, in 1979, the “Environmental Protection Law of the People’s Republic of China (Trial)” mandated that pollutant emissions in excess of the nationally established levels would incur fees based on their quantity and concentration. It also offers a legal foundation for the pollution levy system. In February 1982, the State Council issued comprehensive regulations on the pollution levy’s objective, scope, criteria, extra and reduced conditions, and charge administration. In July 1982, the pollution levy system was formally formed and applied nationwide. In 2003, the total pollutant emission became the basis for the pollution levy system. The pollution levy standards for SO_2_ were increased to 0.63 yuan per kilogram on 1 July 2005. In May 2007, in response to a severe environmental situation, the Chinese central government proposed a binding target of a 10% reduction in the total emissions of major pollutants during the 11th Five-Year Plan period and mandated that the pollution levy standards for SO_2_ be increased from 0.63 yuan per kilogram to 1.26 yuan per kilogram. In reaction to this policy, a number of Chinese provinces have successively modified their pollution levy regulations (refer to Table 1 for more details). The province of Jiangsu assumed the lead on 1 July 2007. In 2008, the provinces of Anhui, Hebei, Shandong, and Inner Mongolia Autonomous all upped their pollution levy standards. In total, 15 provinces in China have upped their pollution charge standards to 1.26 yuan per kilogram as of the end of 2014, with the exception of Beijing, which raised the threshold to 10 yuan per kilogram.

**TABLE 1:** Information on the change of emission fee by regions 2007–2013.

Pilot provinces	Policy change date	Pollution levy rate
Jiangsu	1/07/2007	1.26 yuan/ton
Anhui	1/01/2008	1.26 yuan/ton
Hebei	1/07/2008	1.26 yuan/ton
Shandong	1/07/2008	1.26 yuan/ton
Inner Mongolia	10/07/2008	1.26 yuan/ton
Guangxi	1/01/2009	1.26 yuan/ton
Shanghai	1/01/2009	1.26 yuan/ton
Yunnan	1/01/2009	1.26 yuan/ton
Guangdong	1/04/2010	1.26 yuan/ton
Liaoning	1/08/2010	1.26 yuan/ton
Tianjin	20/12/2010	1.26 yuan/ton
Xinjiang	1/08/2012	1.26 yuan/ton
Beijing	1/01/2014	10 yuan/ton
Ningxia	1/03/2014	1.26 yuan/ton
Ningxia	1/04/2014	1.26 yuan/ton

### Agricultural Root of Collectivism/Individualism in China

Why are some communities collectivistic and others individualistic? The literature on cross-cultural psychology contends that the origins of individualism and collectivism are ecological (Vandello and Cohen, 1999; Nisbett, 2003; Talhelm et al., 2014). Rice-growing regions typically have relatively longer crop-growing seasons, allowing them to double their crop production. Rice responds well to substantial irrigation, dredging, planting, weeding, transplanting, and rigor field leveling. Traditional rice growers met the labor requirements by establishing labor exchanges. Therefore, a history of rice production produces collectivism and increased societal responsibility.

Compared with rice, wheat needs more rainfall and less irrigation. The duration of the wheat-growing season is shorter than the rice-growing season. With additional time, efforts might be dedicated to other endeavors, such as advancing agricultural techniques. Throughout history, women cultivated wheat while men reared livestock. Seasonally, the home men may require to procure water and grass for the herd in remote areas (Moran, 1982; Ang and Fredriksson, 2017). The lower labor input needs also indicate that wheat producers can tend to their own plots with less assistance from other people than rice farmers. Individualists, therefore, embrace human individuality and uniqueness and place a premium on personal ambitions over community goals. In conclusion, wheat production promotes higher individualism, correlated with diminished social responsibility.

## Hypothesis Development

On the one hand, environmental regulation can help companies reduce pollution. On the other hand, as environmental regulations (e.g., environmental taxes and carbon taxes) increase the cost of environmental resources while reducing pollution emissions, firm productivity is frequently impacted negatively. More precisely, when enterprises are subjected to harsh environmental regulations, such as a considerable increase in emission costs, the marginal cost of firm production exceeds the marginal benefit. Controlling firm pollution, in particular, necessitates massive corporate environmental investment. Corporate environmental investments, such as green technology R&D innovation, process improvement, or equipment installation, are typically characterized by lengthy lead times, low upfront returns, and high risk, making it costly for firms to reduce their pollution through pollution abatement investment. Some firms then choose to reduce pollutant emissions by reducing firm production directly.

Individualist vs. collectivist orientations have been demonstrated to influence environmentally conscious behavior. Individuals with collectivistic tendencies are more likely than those with individualistic tendencies to engage in a variety of pro-environmental behaviors, such as resource conservation and green shopping. Moreover, according to a poll performed in New Zealand by Semenova (2015), the more ecologically engaged group (representing sustainable communities) had a more collectivistic value orientation than the less environmentally active group. Environmental activists were more likely to embrace self-transcendent values (similar to collectivism, for example, universalism-concern). Still, non-activists were more likely to endorse self-interest values (similar to individualism, e.g., self-direction). Based on the above theoretical analysis, this study proposes the following research hypothesis:

***H1**: Firms’ pollution emissions decreased dramatically as a result of the increase in emission fees.*
***H2**: Collectivistic CEOs are more likely than individualistic CEOs to take measures to reduce business pollution while maintaining company production in response to the change in environmental policy.*

## Data

We employed four sources of data in our analysis: the Chinese Industrial Firm (CIF) database, the Chinese Industrial Firm Pollution Emission (CIFPE) database, the China Statistical Yearbook, and the Global Agro-Ecological Zones (GAEZ) database. The CIFPE database includes industrial firms that account for 85% of the total emissions of major pollutants in China and comprises information on industrial output, energy input, and pollution emissions. To ensure the integrity and accuracy of these data, they are reported by polluting firms independently, collected by local environmental protection departments, and finally monitored and irregularly checked by environmental protection departments at the county level. The database is considered to hold the most comprehensive and reliable micro-firm pollution emission data in China. The CIF database includes information on all state- and non-state-owned industrial firms that are “above-designated scale” (i.e., output value over 5 million yuan). The total output value of all firms accounts for more than 90% of China’s total industrial output value (Cui et al., 2020). The volume of this database is huge, and it contains rich information on firms.

Based on the method of Brandt et al. (2012), we matched the CIF database and the CIFPE database. First, the two databases were matched year by year, according to the firms’ code; the datasets that had not been matched successfully were matched again according to the firm name. Second, we identified and checked whether the firm in different years is the same firm according to the legal representative’s name, address, postcode, telephone, industry code, main products, and other information. Finally, we processed the data as follows: (1) We eliminated samples that did not meet the Chinese General Accepted Accounting Principles. The deleted samples mainly included firms with fewer than eight employees; samples with total assets less than current assets or net fixed assets; samples with negative current assets, fixed assets, net fixed assets, total sales, or total output; and samples with negative pollutant emissions. (2) The industry code was revised twice, in 2002 and 2011 during the sample period, and it was unified according to the 2002 standard in this study. (3) Due to the inconsistent dates provided for the establishment of some firms, we used the information in the enterprise search^1^ and Baidu search^2^ websites to make corrections.

Culture is typically characterized as the transmission of largely unchanging beliefs and values from one generation to the next by ethnic, religious, and social groups (Guiso et al., 2006). According to Bisin and Verdier (2011), the transmission of cultural values from parents to children is essential in determining an individual’s cultural values. Although individuals may relocate in the future, they carry with them the cultural views and values inherited from their parents or, more generally, from their hometowns. In this way, we measured the collectivism of CEOs based on where they were born. In this study, we defined the collectivism of CEOs using province-level agroecological suitability indices for rice and wheat obtained from agricultural suitability data. The information is derived from the Global Agro-Ecological Zones (GAEZ) database, which was established collaboratively by the International Institute for Applied Systems Analysis (IIASA) and the Food and Agriculture Organization (FAO). Under the assumption that agroecological conditions vary slowly over time, we followed Nunn and Qian (2010) and employed an intermediate level of inputs for the period 1961–2000. The provinces with more rice cultivation area than wheat cultivation area are categorized as collectivistic culturally dominated regions instead of individualistic culturally dominated regions. We then matched the birthplace of our sample CEOs to the agroecological suitability indexes to determine their collectivism. A CEO is considered collectivistic if he or she was born in a collectivistic culturally dominant province.

Finally, this study generates a merged panel dataset at the company level for 2004–2013, which includes data on CEO’s birthplace, firms’ identifying features, manufacturing processes, financial state, and numerous pollutant emissions. We eliminated firms from the final sample if they (1) do not adhere to accounting standards; or (2) have missing or zero pollution emissions during the sample period. Our final sample consists of 9,227 firms and 63,954 observations spanning the years 2004–2013. To minimize the effect of outliers, all variables in this study were winsorized at the 1% level. Table 2 contains a list of the major variables utilized in this study, together with descriptive statistical information about them.

**TABLE 2:** Definition of variables and descriptive statistics.

Variable	Variable definition	Unit	Mean	SD
**Panel A: Pollution measure**				
*SO*_2_	Annual *SO*_*2*_ emissions	Ton	150.391	727.212
*PI*	*SO*_2_ emission intensity: emissions per unit of output	Tons/million $	1.230	2.973
*COD*	Annual COD emissions	Ton	52.058	163.547
**Panel B: Firm characteristics**				
*Output*	Total industrial output	Million dollars	374.583	859.468
*Employee*	Number of persons employed by the firm at the end of the year	Number of employees	561.203	859.921
*Year*	Age of firm	Year	13.484	11.987
*FixedAsset*	Corporate fixed assets	Million dollars	120.716	321.533
*Gearratio*	Gearing ratio: total liabilities/total assets	–	0.5981	0.3041
*Debt*	Credit borrowing: total liability accounts payable	Million dollars	155.430	405.364
*IE*	Interest expenses	Ten thousand dollars	207.566	888.820
**Panel C: CEO characteristics**				
*Collectivism*			0.683	0.857
*Gender*			0.836	0.9563

*Table reports variable definitions and summary statistics of the main variables used in the regression analysis. We winsorized the main variables at the 1 and 99 percentiles to mitigate any undue influences of outliers.*

## Empirical Analysis

### Baseline Results

Since 2007, some provinces in China have gradually increased their emission fees, while others have maintained their current rate. Due to China’s staggered implementation of increased emission fees across provinces and over time, we could utilize the difference-in-difference methodology to determine the causal effect of increased emission fees on corporate pollution reduction.

We can determine the policy effect of an increase in emission charges by comparing the difference between emissions in areas where emission charges were increased before and after the policies in areas where emission charges stayed unchanged. To test the effect of the increased emission fees on corporate pollution reduction, we estimated the following regression model:


(1) $$Y_{j\,t}=\alpha +\beta \,T\,r\,e\,a\,t_{p}\times P\,o\,s\,t_{t}+\lambda \,C\,o\,n\,t\,r\,o\,l_{j\,t}+$$

$$\mu_{t}+\gamma_{i}+\epsilon_{j\,t}$$

Where Y_*jt*_ are the outcome variables, including pollutant emissions, emission intensity, and gross enterprise product, presented in logarithmic form; *j* denotes firm *j*, *p* denotes province *p*, and *t* denotes year *t*; the indicator variable *Treat*_*p*_ × *Post*_*t*_ equals on if province *p* has increased their emission fees in year *t*; otherwise, it is zero. Thus, the coefficient of *Treat*_*p*_ × *Post*_*t*_ provides the difference-in-difference estimate, stating the difference in *Y*_*jt*_ between the provinces that raised and not raised the emission fees. Given the sample period of 2004–2013, the regions where emission fees were increased throughout that time period are selected to be in the treatment group, as illustrated in Table 1, which includes 12 provinces and cities. The control group, which includes 18 provinces and cities, is comprised of places where emission fees were not changed during the sample period of 2004–2013. *Control*_*jt*_ contain firm control variables, which include basic firm information (number of employees and year) and firm operating characteristics (labor productivity, capital-labor ratio, and gearing ratio). In addition, the control variables also include provincial control variables. In the regressions, we also included fixed effects (γ_*i*_) and year fixed effects (μ_*t*_). ϵ_*jt*_ is error term. To address the concerns of autocorrelation among observations associated with a given firm, we clustered standard errors at the city level.

Table 3 represents the estimated results of *SO*_2_ emissions and production of firms affected by the increase in emission fees in regression (1), where columns (1)–(3) do not account for firm characteristics, but columns (4)–(6) do. From columns (4) to (6), it is evident that increasing emission fees significantly reduced *SO*_2_ emissions and firm output by 9.14 and 4.43%, respectively; however, the results also show that firms’ *SO*_2_ emissions per unit of output did not significantly decrease. The findings reveal that enterprises choose to minimize emissions by simply cutting production rather than implementing more environmentally friendly manufacturing practices in reaction to the higher cost of emissions. The results are consistent with the first hypothesis.

**TABLE 3:** Policy effects of emission fee increases.

	(1)	(2)	(3)	(4)	(5)	(6)
						
	*lnSO*_2_	*lnOutput*	*lnPI*	*lnSO*_2_	*lnOutput*	*lnPI*
*Treat*_*p*_ × *Post*_*t*_	−0.0836* (−1.72)	−0.0337* (−1.69)	−0.0871 (−1.48)	−0.0927* (−1.62)	−0.0431** (−2.14)	−0.0732 (−1.55)
*lnOutput*			−0.6131*** (−16.63)			−0.8262*** (−16.16)
*Firm control*	No	No	No	Yes	Yes	Yes
*Firm fixed effects*	Yes	Yes	Yes	Yes	Yes	Yes
*Province control*	Yes	Yes	Yes	Yes	Yes	Yes
*Year fixed effects*	Yes	Yes	Yes	Yes	Yes	Yes
*N*	161,939	173,954	161,939	161,939	173,954	161,939
*R* ^2^	0.732	0.812	0.725	0.793	0.886	0.829

Firm control variables include the number of employees, age, and squared terms; province control variables include GDP per capita, industrial SO_2_ emission intensity, and share of investment in exhaust gas treatment by the province in 2007. Significance: *10%; **5%; ***1%. t-values in parentheses, and they are clustered at the city level.

### Chief Executive Officer Collectivism and Corporate Pollution Behavior

To examine the relationship between CEO’s collectivistic background and corporate pollution behavior after the pollution policy change, we estimated the following OLS regressions:


(2) $$Y_{j\,t}=\alpha +\beta \,T\,r\,e\,a\,t_{p}\times P\,o\,s\,t_{t}+\theta \,T\,r\,e\,a\,t_{p}\times P\,o\,s\,t_{t}\times C\,o\,l\,l\,e\,c\,t\,i\,v\,i\,s\,i\,m_{j\,t}+\,\lambda \,C\,o\,n\,t\,r\,o\,l_{j\,t}+\mu_{t}+\gamma_{i}+\epsilon_{j\,t}$$

The main explanatory variable is *Collectivism*, which is measured by the CEO’s birthplace. Collectivism is a dummy variable that equals one if the CEO was born in a province dominated by rice cultivation and zero if otherwise.

Table 4 reports the regression results for both the collectivist CEO and individualistic CEO firm samples. There is significant heterogeneity in the change of emission fee policy effects after the pollution policy change between collectivistic CEO and individualistic CEO firms. The coefficient of the interaction term *Treat_*p*_ × Post_*t*_ × Collectivism_*jt*_* in the regression shows a significant difference between the two types of firms. Comparing firms with collectivistic CEOs to those with individualistic CEOs, the results indicate a significant drop in *SO*_2_ emissions and *SO*_2_ emissions per corporate output unit. In contrast to the firms with collectivist CEOs, the output of the firms with individualistic CEOs decreased significantly after the policy change. Taken together, our results support the second hypothesis that collectivistic CEOs are more likely than individualistic CEOs to implement strategies to minimize corporate pollution while maintaining firm output in response to a shift in environmental regulation.

**TABLE 4:** Chief executive officer (CEO) collectivism and corporate pollution behavior after the policy change.

	(1)	(2)	(3)
			
	*lnSO*_2_	*lnOutput*	*lnPI*
*Treat*_*p*_ × *Post*_*t*_	−0.0532* (−1.38)	−0.0207* (−1.42)	−0.0368 (−1.21)
*Treat*_*p*_ × *Post*_*t*_ × *Collectivisim*_*jt*_	−0.112*** (3.87)	0.087*** (0.985)	0.133*** (6.98)
*Firm control*	Yes	Yes	Yes
*Firm fixed effects*	Yes	Yes	Yes
*Province control*	Yes	Yes	Yes
*Year fixed effects*	Yes	Yes	Yes
*N*	63,954	63,954	63,954
*R* ^2^	0.657	0.717	0.702

Firm control variables include the number of employees, age, and squared terms; province control variables include GDP per capita, industrial SO_2_ emission intensity, and share of investment in exhaust gas treatment by the province in 2007. Significance: *10%; ***1%. t-values in parentheses, and they are clustered at the city level.

## Heterogeneous Analysis

In the heterogeneous analysis, we explored whether the effect of a CEO’s collectivism is consistent across different types of firms to ensure that the results are not sensitive to sample selection and research design. We split the sample by firm size and firm pollution emission. First, a firm is defined as small if the total assets are below the median for the sample. We then defined a firm as having low pollution emission if its pollution emission is below the median of the sample firms.

Table 5 reports the regression results for the large and small firm samples. The coefficient on *Treat*_*p*_ × *Post*_*t*_ × *Collectivism*_*jt*_ in large firms is not significantly different from that of small firms, suggesting that the results are not sensitive to sample selection based on size. When the sample is divided by pollution emission, the coefficients of *Treat*_*p*_ × *Post*_*t*_ × *Collectivism*_*jt*_ stay comparable in both samples of firms with high and low pollution emissions as shown in Table 6.

**TABLE 5:** Heterogeneity of policy effects between large and small firms.

	(1)	(2)	(3)	(4)	(5)	(6)

	Large firms			Small firms		
		
	*lnSO*_2_	*lnOutput*	*lnPI*	*lnSO*_2_	*lnOutput*	*lnPI*
*Treat*_*p*_ × *Post*_*t*_	−0.0462* (−1.43)	−0.0221* (−1.46)	−0.0329 (−1.26)	−0.0482 (−1.41)	−0.0223** (−1.57)	−0.0316 (−1.24)
*Treat*_*p*_ × *Post*_*t*_ × *Collectivism*_*jt*_	−0.122*** (3.34)	0.072*** (0.839)	0.137*** (6.35)	−0.111*** (3.12)	0.077*** (0.881)	0.131*** (6.13)
*Firm control*	YES	YES	YES	YES	YES	YES
*Firm fixed effects*	YES	YES	YES	YES	YES	YES
*Province control*	YES	YES	YES	YES	YES	YES
*Year fixed effects*	YES	YES	YES	YES	YES	YES
*N*	31,692	31,692	31,692	32,681	32,681	32,681
*R* ^2^	0.612	0.703	0.682	0.631	0.702	0.691

Table reports the regression results of the effect of the emission fee increase on corporate emission behavior for both large and small firms. Columns (1)–(3) are the regression results for the large firm sample, while columns (4)–(6) are the regression results for the small firm sample. Firm control variables include the number of employees, age, and squared terms; province control variables include GDP per capita, industrial SO_2_ emission intensity, and share of investment in exhaust gas treatment by the province in 2007. Significance: *10%; **5%; ***1%. t-values in parentheses, and they are clustered at the city level.

**TABLE 6:** Heterogeneity of policy effects between high emission and low emission firms.

	(1)	(2)	(3)	(4)	(5)	(6)

	High pollution firms			Low pollution firms		
		
	*lnSO*_2_	*lnOutput*	*lnPI*	*lnSO*_2_	*lnOutput*	*lnPI*
*Treat*_*p*_ × *Post*_*t*_	−0.0418* (−1.49)	−0.0283* (−1.31)	−0.0391 (−1.42)	−0.0521 (−1.62)	−0.0207** (−1.38)	−0.0342 (−1.22)
*Treat*_*p*_ × *Post*_*t*_ × *Collectivism*_*jt*_	−0.137*** (3.19)	0.078*** (0.792)	0.128*** (6.72)	−0.125*** (2.92)	0.071*** (0.827)	0.119*** (6.37)
*Firm control*	YES	YES	YES	YES	YES	YES
*Firm fixed effects*	YES	YES	YES	YES	YES	YES
*Province control*	YES	YES	YES	YES	YES	YES
*Year fixed effects*	YES	YES	YES	YES	YES	YES
*N*	31,533	31,533	31,533	32,927	32,927	32,927
*R* ^2^	0.634	0.685	0.648	0.642	0.711	0.636

Table reports the regression results of the effect of the emission fee increase on corporate emission behavior for both large and small firms. Columns (1)–(3) are the regression results for the high pollution firm sample, while columns (4)–(6) are the regression results for the low pollution firm sample. Firm control variables include the number of employees, age, and squared terms; province control variables include GDP per capita, industrial SO_2_ emission intensity, and share of investment in exhaust gas treatment by the province in 2007. Significance: *10%; **5%; ***1%. t-values in parentheses, and they are clustered at the city level.

## Conclusion and Policy Implication

Climate change, commonly described as the “ultimate commons problem,” is caused by anthropogenic glasshouse gas (GHG) emissions such as CO_2_ and is anticipated to have severe ecological and economic consequences. The industrial sector is a major contributor to global glasshouse gas emissions. Together with primary industries, the industrial sector is responsible for roughly 40% of worldwide glasshouse gas emissions. In this study, we evaluated the impact of the CEO’s collectivistic background on the firm’s pollution abatement behavior using the agricultural root of the CEO’s birthplace as a measure of collectivism. We found that as a result of the increased emission prices, firms’ pollutant emissions reduced considerably. We also found that, in reaction to a change in environmental policy, collectivistic CEOs are more likely than individualistic CEOs to take action to reduce company pollution while maintaining firm production. The results are unaffected by the sample firms’ size or pollutant emission volume.

Overall, our research adds to the knowledge of the value of a CEO’s intrinsic characteristics by shedding fresh light on the relationship between personality traits and corporate pollution abatement behavior. More crucially, we showed that corporate pollution abatement behavior is heavily influenced by culture. Our research highlights the importance of culture and has significant implications for future research into the relationship between culture and corporate behavior.

## Data Availability Statement

Publicly available datasets were analyzed in this study. This data can be found here: The Chinese Industrial Firm (CIF) Database, the Chinese Industrial Firm Pollution Emission (CIFPE) Database.

## Author Contributions

All authors listed have made a substantial, direct, and intellectual contribution to the work, and approved it for publication.

## Conflict of Interest

The authors declare that the research was conducted in the absence of any commercial or financial relationships that could be construed as a potential conflict of interest.

## Publisher’s Note

All claims expressed in this article are solely those of the authors and do not necessarily represent those of their affiliated organizations, or those of the publisher, the editors and the reviewers. Any product that may be evaluated in this article, or claim that may be made by its manufacturer, is not guaranteed or endorsed by the publisher.

## Footnotes

1
https://www.qcc.com/

2
https://www.baidu.com/
