# Corrigendum: Chief executive officer collectivism and corporate pollution abatement behavior: Evidence from industrial firms in China

**DOI:** 10.3389/fpsyg.2022.1009891

**Published:** 2022-08-19

**Authors:** Shumin Wang, Yikun Huang, Chao Zhong, Boxi Li

**Affiliations:** ^1^Research Center for Economy of Upper Reaches of the Yangtse River, Chongqing Technology and Business University, Chongqing, China; ^2^School of Public Affairs Administration, China Agricultural University, Beijing, China; ^3^Business School, Beijing Normal University, Beijing, China; ^4^China Resources Environment Carbon Neutrality Research Programme, Beijing, China

**Keywords:** environmental regulation, climate change, financing constraints, pollution reduction, CEO collectivism

In the published article, there was an error in the affiliation of author Chao Zhong. Chao Zhong was listed as “Chao Zhong^3, 4^” (“3 Institute of European Studies, Chinese Academy of Social Sciences, Beijing, China; 4 Business School, Beijing Normal University, Beijing, China”) However affiliation 3 was incorrectly included. Chao Zhong has been amended to only “Chao Zhong^3^” (“3 Business School, Beijing Normal University, Beijing, China”).

The authors apologize for this error and state that this does not change the scientific conclusions of the article in any way. The original article has been updated.

## Publisher's note

All claims expressed in this article are solely those of the authors and do not necessarily represent those of their affiliated organizations, or those of the publisher, the editors and the reviewers. Any product that may be evaluated in this article, or claim that may be made by its manufacturer, is not guaranteed or endorsed by the publisher.

